# Excess Mortality With Alzheimer Disease and Related Dementias as an Underlying or Contributing Cause During the COVID-19 Pandemic in the US

**DOI:** 10.1001/jamaneurol.2023.2226

**Published:** 2023-07-17

**Authors:** Ruijia Chen, Marie-Laure Charpignon, Rafeya V. Raquib, Jingxuan Wang, Erika Meza, Hélène E. Aschmann, Michelle A. DeVost, Alyssa Mooney, Kirsten Bibbins-Domingo, Alicia R. Riley, Mathew V. Kiang, Yea-Hung Chen, Andrew C. Stokes, M. Maria Glymour

**Affiliations:** 1 Department of Epidemiology and Biostatistics, University of California, San Francisco, San Francisco; 2 Institute for Data, Systems, and Society, Massachusetts Institute of Technology, Cambridge; 3 Department of Global Health, Boston University School of Public Health, Boston, Massachusetts; 4 Institute for Health Policy Studies, University of California, San Francisco, San Francisco; 5 Department of Medicine, University of California, San Francisco, San Francisco; 6 Editor in Chief, *JAMA*; 7 Department of Sociology, University of California, Santa Cruz, Santa Cruz; 8 Department of Epidemiology and Population Health, Stanford University School of Medicine, Stanford, California; 9 Department of Epidemiology, Boston University School of Public Health, Boston, Massachusetts

## Abstract

**Importance:**

Adults with Alzheimer disease and related dementias (ADRD) are particularly vulnerable to the direct and indirect effects of the COVID-19 pandemic. Deaths associated with ADRD increased substantially in pandemic year 1. It is unclear whether mortality associated with ADRD declined when better prevention strategies, testing, and vaccines became widely available in year 2.

**Objective:**

To compare pandemic-era excess deaths associated with ADRD between year 1 and year 2 overall and by age, sex, race and ethnicity, and place of death.

**Design, Setting, and Participants:**

This time series analysis used all death certificates of US decedents 65 years and older with ADRD as an underlying or contributing cause of death from January 2014 through February 2022.

**Exposure:**

COVID-19 pandemic era.

**Main Outcomes and Measures:**

Pandemic-era excess deaths associated with ADRD were defined as the difference between deaths with ADRD as an underlying or contributing cause observed from March 2020 to February 2021 (year 1) and March 2021 to February 2022 (year 2) compared with expected deaths during this period. Expected deaths were estimated using data from January 2014 to February 2020 fitted with autoregressive integrated moving average models.

**Results:**

Overall, 2 334 101 death certificates were analyzed. A total of 94 688 (95% prediction interval [PI], 84 192-104 890) pandemic-era excess deaths with ADRD were estimated in year 1 and 21 586 (95% PI, 10 631-32 450) in year 2. Declines in ADRD-related deaths in year 2 were substantial for every age, sex, and racial and ethnic group evaluated. Pandemic-era ADRD-related excess deaths declined among nursing home/long-term care residents (from 34 259 [95% PI, 25 819-42 677] in year 1 to −22 050 [95% PI, −30 765 to −13 273] in year 2), but excess deaths at home remained high (from 34 487 [95% PI, 32 815-36 142] in year 1 to 28 804 [95% PI, 27 067-30 571] in year 2).

**Conclusions and Relevance:**

This study found that large increases in mortality with ADRD as an underlying or contributing cause of death occurred in COVID-19 pandemic year 1 but were largely mitigated in pandemic year 2. The most pronounced declines were observed for deaths in nursing home/long-term care settings. Conversely, excess deaths at home and in medical facilities remained high in year 2.

## Key Points

**Question:**

How did mortality with Alzheimer disease and related dementias (ADRD) as an underlying or contributing cause change during the COVID-19 pandemic?

**Findings:**

This cross-sectional study found a large increase in ADRD-related mortality during pandemic year 1 (March 2020 to February 2021) compared with prepandemic mortality rates but substantial declines from year 1 to year 2 (March 2021 to February 2022). The largest declines occurred in nursing home/long-term care settings, but excess mortality occurring at home and medical facilities remained high in year 2.

**Meaning:**

Major reductions in pandemic-era ADRD-related excess mortality were achieved in pandemic year 2 for nursing home/long-term care settings residents.

## Teaser

This cross-sectional study compares COVID-19 pandemic-era excess deaths associated with Alzheimer disease or related dementias between year 1 and year 2 overall and by age, sex, race and ethnicity, and place of death.

## Introduction

Older adults with Alzheimer disease and related dementias (ADRD) are particularly vulnerable to the direct and indirect impacts of the COVID-19 pandemic.^1^ Individuals with ADRD may have difficulty adopting behavioral changes to reduce infection risk or regulating contact in high-risk settings. Common comorbidities among older adults with ADRD increase the risk of death if they are infected with SARS-CoV-2.^2,3^ Social isolation resulting from COVID-19 lockdowns may exacerbate depression and loneliness among individuals with ADRD, increasing their risk of hospitalization and mortality.^4,5^ Disruptions in care and services may have disproportionately affected individuals with ADRD.^6^ In the early pandemic period, excess mortality disproportionately affected older adults with ADRD in long-term care facilities, likely due to the high prevalence of comorbidities among residents, staffing shortages, isolation from family members and/or other essential caregivers, and challenges in implementing and enforcing infection prevention and control measures.^1^ Sex and racial and ethnic disparities in excess mortality from ADRD were also noted, with more excess deaths in females (vs males) and non-Hispanic Black and Hispanic (vs non-Hispanic White) older adults in the early pandemic.^1,7^

While research has documented substantial excess mortality among individuals with ADRD in the early pandemic, it is unclear how its magnitude changed as the pandemic evolved.^1^ Pharmaceutical and nonpharmaceutical preventive measures became widely available in pandemic year 2, but vaccine distribution and uptake of other measures were inconsistent.^8,9^ Tracking excess mortality associated with ADRD over the pandemic is critical to inform policy and research priorities, as changes in mortality provide insights into whether current preventive measures are effective at protecting older adults with ADRD. This study assessed pandemic-era changes in mortality with ADRD as an underlying or contributing cause comparing the COVID-19 pandemic year 1 (March 2020 to February 2021) to year 2 (March 2021 to February 2022), in the overall population and by age, sex, race and ethnicity, and place of death.

## Methods

### Data

Final death certificate data from January 2014 to December 2021 and provisional death certificate data from January 2022 to February 2022 were extracted from the National Center for Health Statistics mortality surveillance system.^10,11^ We considered deaths with any mention of ADRD on the death certificate, including as the underlying cause or any of up to 19 listed contributing conditions as deaths with ADRD. ADRD was classified by *International Statistical Classification of Diseases and Related Health Problems, Tenth Revision *codes and included unspecified dementia, Alzheimer disease, vascular dementia, and other degenerative diseases of the nervous system (see eTable 1 in Supplement 1 for a listing of codes). Throughout this article, we labeled these as *ADRD-related deaths* for parsimony. We considered deaths among adults aged 65 years and older because this population accounts for most ADRD-related mortality.^10,12^ We obtained age-, sex-, race and ethnicity–, and state-specific population estimates from the US Census Bureau Population Estimates Program. We calculated excess mortality rates in 2020 and 2021 using the corresponding year’s July population estimates as the denominator. Since the 2022 population estimate was not yet available at the time of manuscript preparation, we used the July 2021 population estimates as the denominators to calculate death rates in January and February 2022. This study used publicly available data and was not subject to human subjects review at University of California, San Francisco and followed the Strengthening the Reporting of Observational Studies in Epidemiology (STROBE) reporting guideline.

The National Center for Health Statistics provided bridged-race death data from 2014 to 2020 and single-race death data from 2018 to 2022. We combined 2014-2017 bridged-race data and 2018-2022 single-race data to estimate race-specific excess mortality. To evaluate the bias associated with combining single- and bridged-race data, we compared both numerators (death counts) and denominators (population size) using single- vs bridged-race categories for the period during which both data sources were available, ie, 2018-2020 (eFigure 1 in Supplement 1). Differences between the 2 data sources in both numerators and denominators were very small for all groups except American Indian and Alaska Native individuals. As such, we did not include American Indian and Alaska Native decedents.

### Exposures and Stratification Variables

COVID-19 pandemic year 1 was defined as March 2020 through February 2021; year 2 was defined as March 2021 through February 2022. We considered 4 waves of the pandemic based on the dominant viral variant: early pandemic (March 1, 2020, to September 31, 2020), Alpha wave (October 1, 2020, to June 30, 2021), Delta wave (July 1, 2021, to November 30, 2021), and Omicron wave (December 1, 2021, to February 28, 2022).

Additional stratification variables included age (65-74, 75-84, ≥85 years), sex (male and female), race and ethnicity (Hispanic; non-Hispanic Asian or Pacific Islander; non-Hispanic Black; non-Hispanic White), and place of death (medical facilities, including inpatient, outpatient, emergency department, dead on arrival; decedent’s home; nursing home/long-term care [NH/LTC]; or hospice facility).

### Statistical Analysis

We defined pandemic-era excess ADRD-related deaths as the difference between observed and expected deaths over the same period. To estimate expected deaths, we fitted auto-regressive integrated moving-average models (ARIMA) to monthly ADRD-related death count data from January 2014 to February 2020. We used this prepandemic prediction model to forecast the number of ADRD-related deaths that would have been expected in the absence of the pandemic, accounting for historical mortality trends and seasonality.^13^ We selected the model with the lowest Akaike information criterion and calculated monthly excess deaths as observed minus expected deaths. We calculated total excess deaths by summing all monthly excess deaths and calculated corresponding 95% prediction intervals (PIs) by simulating the expected death model 10 000 times, selecting the 2.5 and 97.5 percentiles, and subtracting them from the number of observed deaths. We calculated risk ratios as the observed number of deaths divided by the expected number of deaths. We calculated excess mortality rates per capita, as the number of excess deaths divided by the corresponding population size and multiplied by 100 000.

We performed stratified analyses by age, sex, race and ethnicity, and place of death. To facilitate comparisons across groups, excess deaths were age standardized in 10-year categories to the 2000 population when applicable.

### Sensitivity and Exploratory Analyses

To validate the performance of the ARIMA model, we performed sensitivity analyses using data from 2014 to 2018 to forecast monthly death rates from January to December 2019. All observed deaths fell within the 95% PI of the expected deaths, suggesting accurate predictions (eTable 2 in Supplement 1). In an additional sensitivity analysis, we imputed single-race monthly death data for 2014-2017 bridged-race monthly death data (eTable 3 in Supplement 1). To understand whether excess ADRD-related deaths were primarily due to SARS-CoV-2 infections, we cross-classified ADRD deaths and COVID-19 deaths into 4 groups and analyzed the corresponding trends: (1) ADRD as an underlying cause and COVID-19 as a contributing cause; (2) ADRD as an underlying cause and COVID-19 not listed as a contributing cause; (3) COVID-19 as an underlying cause and ADRD as a contributing cause; and (4) neither COVID-19 nor ADRD as an underlying cause (eg, cancer deaths) but ADRD as a contributing cause.

We conducted exploratory analyses to investigate potential reasons for notable declines in excess deaths between years 1 and 2. First, we evaluated how changes in the number of NH/LTC residents during the pandemic might have affected the estimation of excess deaths in NH/LTC settings. To this end, we obtained the monthly number of NH/LTC residents from the Centers for Medicare & Medicaid Services (CMS) for 2019 to 2022. Specifically, we adjusted the observed number of deaths, accounting for the relative change in the population size of NH/LTC residents by comparing each month between March 2020 to February 2022 to the corresponding month in 2019 (eAppendix 1 in Supplement 1).^14^ Second, we evaluated trends in other causes of death at home or in NH/LTCs to investigate the possibility of frailty selection, ie, the frailest individuals with ADRD would have died in pandemic year 1, leaving an unusually small number of individuals with ADRD at risk of death in year 2. We compared the temporal patterns of ADRD-related deaths with deaths from cancer, heart disease, respiratory disease, and cerebrovascular disease. To facilitate comparisons, we defined a baseline monthly mortality level for each cause, estimated as the mean death count over the 12 months preceding the pandemic (March 2019-February 2020). Third, we used weekly COVID-19 vaccination data from the US Centers for Disease Control and Prevention COVID Data Tracker to understand how vaccination may have affected excess mortality. Specifically, we assessed how changes in annualized excess ADRD-related deaths differed by vaccine coverage across states (eAppendix 2 in Supplement 1).^15^ All analyses were performed using R version 4.2.1 (R Foundation).

## Results

**Figure 1.**
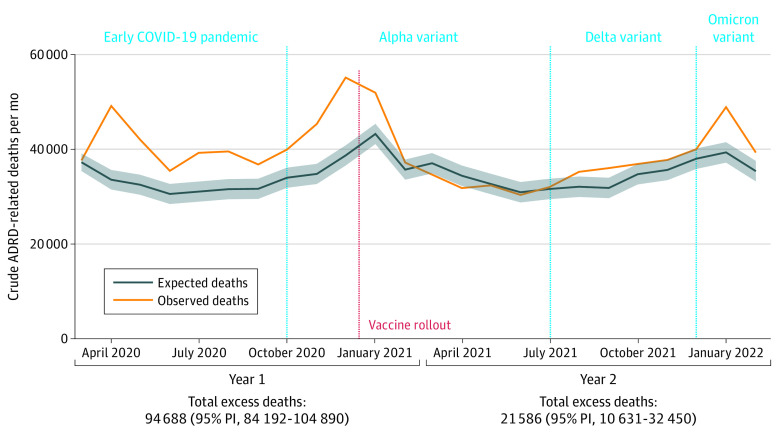
Crude COVID-19 Pandemic-Era Excess Deaths With Alzheimer Disease and Related Dementias (ADRDs) Listed as an Underlying or Contributing Cause Between March 2020 and February 2022 in the US Death data were obtained from the US Centers for Disease Control and Prevention WONDER Multiple Cause of Death Files. Excess deaths were defined as the difference between the observed number of deaths (orange) and the expected number of deaths (blue). The red dashed line indicates the start of vaccine rollouts. Predictions were based on best-fit auto-regressive integrated moving-average models. Shaded areas indicate 95% prediction intervals (PIs).

In the COVID-19 pandemic year 1, there were 509 179 ADRD-related deaths among individuals 65 years and older. Based on prepandemic death rates, 414 491 (95% PI, 404 289-424 987) ADRD-related deaths were expected in year 1, implying a year-1 excess of 94 688 deaths (95% PI, 84 192-104 890). In year 2, ADRD-related deaths declined to 435 156, implying an excess of 21 586 deaths (95% PI, 10 631-32 450) (Figure 1). The pandemic-era excess in ADRD-related deaths thus declined by 77% from year 1 to year 2. In year 2, there was a decline in the ADRD-related deaths both with COVID-19 (n = 45 761) and without COVID-19 (n = 28 245) listed as an underlying or contributing cause (eFigure 2 in Supplement 1).

Pandemic-era excess ADRD-related death rates declined substantially between the first and second year of the pandemic for every age, sex, and racial and ethnic group evaluated (Table and eTable 3 in Supplement 1).

**Table.** Observed, Estimated Expected, and Estimated Excess Mortality Among Older Adults With Alzheimer Disease and Related Dementias (ADRDs) in Pandemic Year 1 Compared With Pandemic Year 2 in the US, Overall and by Age, Sex, and Racial and Ethnic Identity^a^

Characteristic	Pandemic year 1^b^				Pandemic year 2^c^			
	ADRD-related deaths per 100 000 population, No.			Observed:expected ratio of ADRD-related deaths in pandemic year 1 (95% PI)	ADRD-related deaths per 100 000 population, No.			Observed:expected ratio of ADRD-related deaths in pandemic year 2 (95% PI)
	Observed	Expected based on prepandemic death rates (95% PI)	Excess during pandemic year 1 (95% PI)		Observed	Expected based on prepandemic death rates (95% PI)	Excess during pandemic year 2 (95% PI)	
All	1050	836 (806 to 869)	214 (181 to 244)	1.26 (1.21 to 1.30)	892	848 (812 to 885)	42 (7 to 80)	1.05 (1.01 to 1.10)
Age group, y								
65-74	126	98 (97 to 100)	28 (26 to 29)	1.28 (1.26 to 1.30)	112	98 (96 to 100)	14 (12 to 15)	1.14 (1.12 to 1.15)
75-84	922	718 (697 to 739)	204 (184 to 225)	1.28 (1.25 to 1.32)	793	711 (687 to 735)	82 (58 to 105)	1.11 (1.08 to 1.15)
≥85	5352	4319 (4121 to 4534)	1033 (819 to 1231)	1.24 (1.18 to 1.30)	4503	4435 (4202 to 4687)	68 (−184 to 301)	1.02 (0.96 to 1.07)
Sex								
Female	1108	871 (835 to 910)	237 (199 to 273)	1.27 (1.22 to 1.33)	942	878 (836 to 924)	63 (17 to 105)	1.07 (1.02 to 1.13)
Male	947	770 (753 to 787)	177 (160 to 194)	1.23 (1.20 to 1.26)	807	769 (751 to 786)	37 (21 to 56)	1.05 (1.03 to 1.07)
Racial and ethnic identity								
Asian	570	431 (418 to 445)	139 (125 to 152)	1.32 (1.28 to 1.36)	465	425 (411 to 440)	40 (25 to 54)	1.09 (1.06 to 1.13)
Black	1140	843 (826 to 863)	297 (278 to 314)	1.35 (1.32 to 1.38)	916	830 (811 to 853)	86 (63 to 105)	1.10 (1.07 to 1.12)
Hispanic	847	625 (614 to 637)	222 (210 to 233)	1.36 (1.33 to 1.38)	690	618 (606 to 631)	72 (59 to 84)	1.12 (1.09 to 1.14)
White	1096	907 (876 to 938)	189 (158 to 220)	1.21 (1.17 to 1.25)	945	909 (874 to 943)	36 (2 to 71)	1.04 (1.00 to 1.08)

Abbreviation: PI, prediction interval.

^a^ Death data were obtained from the US Centers for Disease Control and Prevention WONDER Multiple Cause of Death Files. Population estimates were obtained from the US Census Bureau Population Estimates Program. Death certificates with any mention of ADRD were included. All analyses were restricted to aged 65 years and older.

^b^ COVID-19 pandemic year 1 indicates March 2020 to February 2021.

^c^ COVID-19 pandemic year 2 indicates March 2021 to February 2022.

Year 2 of the pandemic saw major declines in ADRD-related deaths occurring in NH/LTCs (eTable 4 in Supplement 1) from 34 259 (95% PI, 25 819-42 677) crude excess deaths in year 1 to −22 050 (95% PI, −30 765 to −13 273) excess deaths in year 2. In contrast, in year 2, there were only modest declines in ADRD-related deaths occurring at home (from 34 487 [95% PI, 32 815-36 142] to 28 804 [95% PI, 27 067-30 571]).

**Figure 2.**
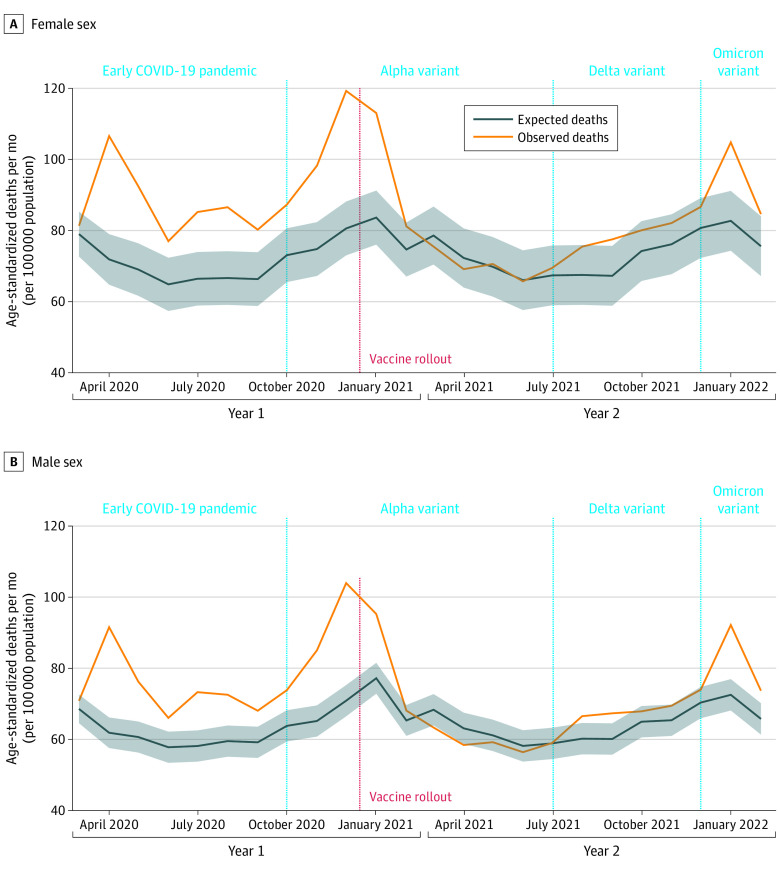
Age-Adjusted Per-Capita Excess Deaths Associated With Alzheimer Disease and Related Dementias Between March 2020 and February 2022 in the US by Sex We show the age-standardized monthly excess deaths (95% prediction interval) per 100 000 population by sex. Death data were obtained from the US Centers for Disease Control and Prevention WONDER Multiple Cause of Death Files. Population estimates were obtained from the US Census Bureau Population Estimates Program and Centers for Disease Control and Prevention WONDER. Excess deaths were defined as the difference between the observed number of deaths (orange) and the expected number of deaths (blue). The red dashed line indicates the start of vaccine rollouts (mid-December 2020). Predictions were based on best-fit auto-regressive integrated moving-average models. Shaded areas indicate 95% prediction intervals.

The patterns of changes in monthly ADRD-related mortality were similar for male and female individuals, with substantial declines following vaccination rollouts in mid-December 2020. The number of observed deaths continued to decline during the Alpha wave, increased during the Delta wave, and increased substantially during the Omicron wave (Figure 2).

**Figure 3.**
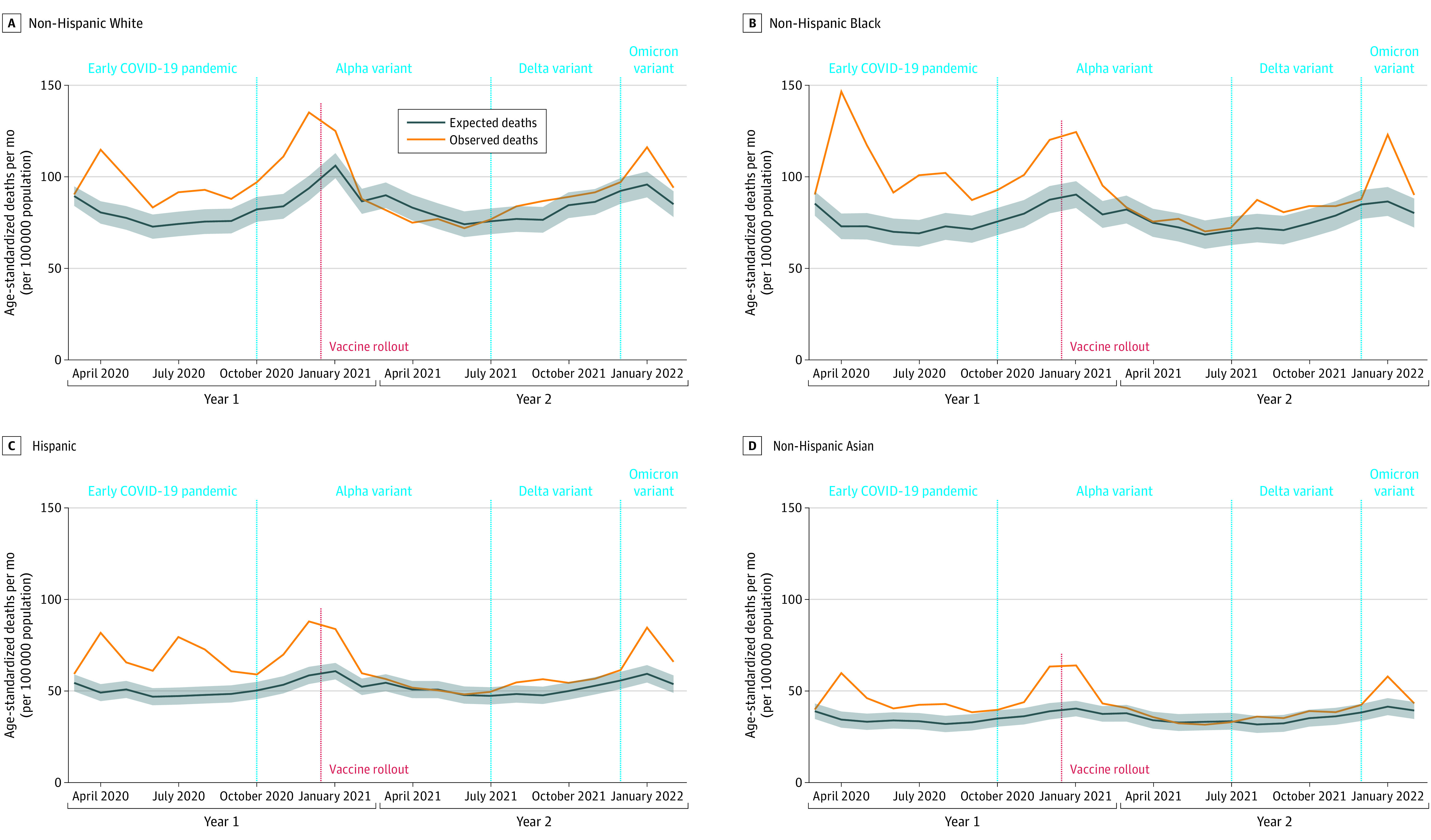
Age-Adjusted Per-Capita Excess Deaths Associated With Alzheimer Disease and Related Dementias Between March 2020 and February 2022 in the US by Race and Ethnicity Death data were obtained from the US Centers for Disease Control and Prevention WONDER Multiple Cause of Death Files. Population estimates were obtained from the US Census Bureau Population Estimates Program and Centers for Disease Control and Prevention WONDER. Excess deaths were defined as the difference between the observed number of deaths (orange) and the expected number of deaths (blue). Predictions were based on best-fit auto-regressive integrated moving-average models. The red dashed line indicates the start of vaccine rollouts. Shaded areas indicate 95% prediction intervals.

The early pandemic was characterized by large racial and ethnic disparities in excess deaths. In April 2020, excess ADRD-related deaths per 100 000 persons were 25 (95% PI, 21-30) for Asian adults, 64 (95% PI, 58-70) for Black adults, 33 (95% PI, 28-37) for Hispanic adults, and 30 (95% PI, 24-35) for White adults (Figure 3). After the mortality peak late in pandemic year 1 and the beginning of vaccine rollout, ADRD-related deaths decreased for all racial and ethnic groups.

**Figure 4.**
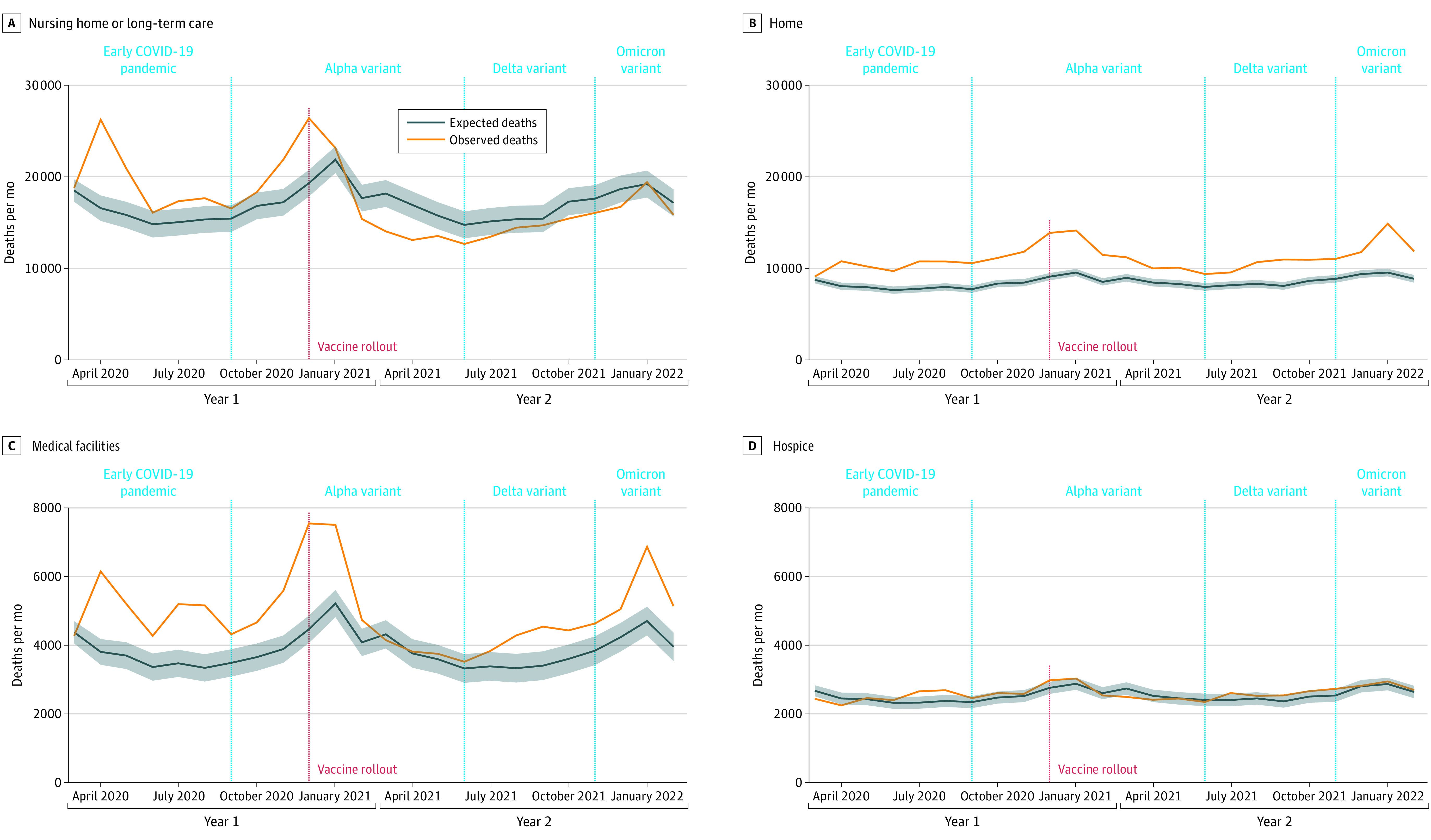
Crude Excess Deaths Associated With Alzheimer Disease and Related Dementias Between March 2020 and February 2022 in the US by Place of Death Death data were obtained from the US Centers for Disease Control and Prevention WONDER Multiple Cause of Death Files. Excess deaths were defined as the difference between the observed number of deaths (orange) and the expected number of deaths (blue). The red dashed line indicates the start of vaccine rollouts. Predictions were based on best-fit auto-regressive integrated moving-average models. Death rates were not age adjusted because we did not have information about the population size in each place of death.

Long-term care facilities were hard hit in the early pandemic (Figure 4). After vaccine rollout began, ADRD-related deaths in NH/LTC settings declined significantly, with lower than expected deaths throughout the Delta wave and most of the Omicron wave. In contrast, ADRD-related deaths occurring at home remained high throughout year 2 of the pandemic, even after vaccine rollout began. ADRD-related deaths in medical facilities fell after vaccine rollout but increased during the Delta wave.

We investigated potential reasons for declines in excess deaths. First, our results about excess mortality in NH/LTC settings during year 2 were robust to pandemic-period changes in the size of the NH/LTC population, relative to the reference year of 2019. Accounting for changes in the number of NH/LTC residents reported to CMS did not materially alter our results: after adjusting for population size change, the number of excess deaths in NH/LTC settings fell to an estimated 34 149 (95% PI, 25 709-42 567) in year 1 and an estimated −2914 (95% PI, −11 628 to 5863) in year 2. Furthermore, the overall trends in the adjusted and unadjusted monthly death counts were similar (eFigure 3 in Supplement 1). Second, comparing across causes of death in NH/LTCs, we found ADRD-related mortality increased from the prepandemic average by 60% in year 1 but only 18% in year 2; patterns were similar for other causes of death that move in tandem with COVID-19, including heart (51% elevation in year 1 and 12% elevation in year 2), respiratory (31% in year 1 and 10% in year 2), and cerebrovascular diseases (35% in year 1 and 18% in year 2) compared with their respective 12-month prepandemic baselines. In contrast, cancer-related deaths occurring in NH/LTCs remained consistently below historical levels (77% of prepandemic levels in year 1 and 82% of prepandemic levels in year 2) (eFigure 4 in Supplement 1). Third, in states in the highest tertiles of pandemic year 1 excess deaths, reductions in pandemic-era excess ADRD-related mortality in year 2 were correlated with both vaccination coverage (*r* = −0.54 [95% CI, −0.08 to −0.81]) and velocity (*r* = −0.67 [95% CI, −0.87 to −0.29]) (eFigure 5 in Supplement 1). These patterns of correlation were not observed in states in the lowest tertile of excess deaths in year 1.

## Discussion

Using a national data set, we found a large excess in ADRD-related deaths in COVID-19 pandemic year 1, which declined significantly in pandemic year 2. Declines in excess mortality occurred for every age, sex, and racial and ethnic group examined. ADRD-related deaths in NH/LTC settings accounted for most of the decline, whereas ADRD-related excess deaths that occurred at home and in medical facilities remained high throughout the pandemic.

People living with ADRD were uniquely vulnerable to the pandemic. Our findings are consistent with prior research showing large adverse effects of the early pandemic on older adults with ADRD.^1,16^ For example, a Medicare-based study showed that in 2020, all-cause mortality was 26% higher than expected in people with ADRD compared with prior years.^1^ Evaluating whether ADRD-related deaths declined in the second year of the pandemic gives insight into whether people with ADRD are benefiting from the evidence and technologies for prevention and treatment developed over the first year of the pandemic, including vaccination. Across all groups and place-of-death settings, we observed a significant decline in excess deaths during the Alpha wave (October 1, 2020, to June 30, 2021), in contrast with a pronounced increase during the Delta wave (July 1, 2021, to November 31, 2021). These divergent patterns across waves may reflect not only changes in public health policies and interventions but also differences in transmissibility, infectivity, and mortality rates among COVID-19 variants.

ADRD-related deaths fell in pandemic year 2, primarily due to reductions in deaths in NH/LTC facilities. This finding cannot be explained by declines in the number of NH/LTC residents as the overall trend remained similar after we accounted for changes in population size using the CMS data. The frailty selection hypothesis alone is not sufficient to explain the results. If the frailty selection hypothesis is supported, we would expect to see reductions in all leading causes of death. However, cancer deaths in nursing homes remained similar between years 1 and 2. Our finding that faster vaccine rollout and greater coverage were associated with larger reductions in ADRD-related deaths in year 2 suggests that access to vaccines, both for persons living with ADRD and their care professionals, may play a key role in reducing excess deaths. Vaccination among staff and residents may lower excess deaths in nursing homes directly through preventing viral transmission and infection and indirectly through reducing social isolation.^17^ In year 2, several initiatives to reduce SARS-CoV-2 transmission implemented in NH/LTC facilities may have further contributed to declines in excess mortality in these settings, including limiting the use of shared spaces and improved surveillance testing of staff members.^18^

Relatedly, the persistently high levels of ADRD-related deaths occurring at home suggest that community-dwelling older adults with ADRD may not have benefited similarly from COVID-19 preventive measures. Community-dwelling older adults with dementia have been found to be more likely to put off care during the pandemic than those who live in nursing homes, potentially contributing to high-excess home deaths.^19^ They have also been found to have higher mortality following a COVID-19 diagnosis than their counterparts without dementia, and the disparities persisted in 2021 despite the availability of vaccines.^20^ Increased policy efforts are imperative for reducing excess deaths among community-dwelling older adults.

Research conducted prior to the pandemic has yielded inconsistent results on racial and ethnic disparities in ADRD mortality.^21,22,23,24,25^ While we could not directly assess racial and ethnic disparities in ADRD-related mortality without population data on the number of individuals living with ADRD by race and ethnicity, our analysis of mortality differences by race and ethnicity highlights concerning patterns in excess ADRD-related deaths during the pandemic. Our findings for year 1 suggest that Black older adults with ADRD experienced substantially higher excess deaths in the early pandemic. Due to the racial segregation of nursing home facilities, Black individuals with ADRD may be more likely to cluster in nursing homes that have worse infection rates.^26,27,28^ Discrimination in medical settings and differential prevalence of comorbidities may also have contributed to this excess mortality. Racial and ethnic disparities in excess ADRD-related deaths persisted despite declines in ADRD-related deaths across all racial and ethnic groups in year 2. These findings highlight the need to monitor inequalities and attend to how structural racism can exacerbate vulnerability to ADRD-related and COVID-19 mortality.

### Limitations and Strengths

Our data may not fully capture deaths among all individuals with ADRDs, if ADRD was undiagnosed, or if the diagnosis was not considered to contribute to death.^29^ However, the patterns of temporal changes in ADRD-related excess deaths that our study revealed are corroborated by Medicare data, which showed a decline in excess deaths among individuals with ADRD in 2021.^30^ Our findings may underestimate the number of ADRD-related deaths from racial and ethnic minority groups in whom ADRD underdiagnosis is common.^31^ Finally, the 2022 death certificate data are provisional. Although analyzing finalized death certificates would be ideal, using currently available data is critical to inform timely policy responses.

This study has several strengths. The inclusion of 2020, 2021, and early 2022 data allowed us to capture the dynamics of the pandemic’s impact on individuals who lived with ADRD. Most prior research relied on underlying cause of death classification.^7,16^ Our inclusion of both the underlying and contributing causes of death likely lessens the impact of misclassification of both ADRD and COVID-19.^32,33^ Our time series models starting in 2014 accounted for prepandemic temporal trends in deaths, for example due to population aging or increased ADRD diagnoses.^34^

## Conclusions

COVID-19 pandemic-era mortality with ADRD as an underlying or contributing cause has been extremely high. These deaths are often preventable, and ADRD-related mortality fell markedly in NH/LTC settings later in the pandemic. Vaccinations were likely critical to these improvements. Our findings underscore the urgent need to mitigate the pandemic’s impacts on community-dwelling older adults with ADRD.

## Notes

Supplement 1. eAppendix 1. Asymptotic maximum coverage rate (K) and vaccination velocity estimation eAppendix 2. Adjusted Observed Deaths Based on Changes in the Number of Nursing Home Resident Before and After the COVID-19 Pandemic Lockdown eTable 1. Alzheimer’s Disease or Related Dementias ICD Codes eTable 2. Observed and Estimated Expected Per-Capita Deaths Associated with Alzheimer’s Disease or Related Dementias Between January and December 2019 eTable 3. Observed, Estimated Expected, and Estimated Excess Mortality Associated with Alzheimer’s Disease or Related Dementias from March 2020 to February 2022 in the United States, Stratified by Racial and Ethnic Identity (“imputed” single-race monthly death data for 2014-2017 bridged-race data) eTable 4. Observed, Estimated Expected, and Estimated Crude Excess Mortality Associated with Alzheimer’s Disease or Related Dementias from March 2020 to February 2022 in the United States, Stratified by Place of Death eFigure 1. Percent Differences in the Number of ADRD Deaths (left panel) and Population Sizes (right panel) Between Single-Race vs. Bridged-Race Data (2018-2020) eFigure 2. ADRD Death Subgroups eFigure 3. Observed, Expected, and Adjusted Observed Death in nursing home/long-term care facilities between 2020/03 and 2022/02, adjusted for percentage changes in population size eFigure 4. Changes in the number of monthly deaths from leading causes of death between 2020/03 and 2022/02 eFigure 5. Changes in State-level Crude Excess Deaths Associated with Alzheimer’s Disease or Related Dementias between Year 1 and Year 2 in the United States, by Maximum Vaccination Coverage Rate and Vaccination Velocity

Supplement 2. Data sharing statement
