# Supplementary material for: Excess Mortality With Alzheimer Disease and Related Dementias as an Underlying or Contributing Cause During the COVID-19 Pandemic in the US
Source: JAMA Neurol. 2023 Jul 17;80(9):919–28. doi: 10.1001/jamaneurol.2023.2226 (PMC10352932; doi:10.1001/jamaneurol.2023.2226)
Supplement: Supplement 1. — eAppendix 1. Asymptotic maximum coverage rate (K) and vaccination velocity estimation eAppendix 2. Adjusted Observed Deaths Based on Changes in the Number of Nursing Home Resident Before and After the COVID-19 Pandemic Lockdown eTable 1. Alzheimer’s Disease or Related Dementias ICD Codes eTable 2. Observed and Estimated Expected Per-Capita Deaths Associated with Alzheimer’s Disease or Related Dementias Between January and December 2019 eTable 3. Observed, Estimated Expected, and Estimated Excess Mortality Associated with Alzheimer’s Disease or Related Dementias from March 2020 to February 2022 in the United States, Stratified by Racial and Ethnic Identity (“imputed” single-race monthly death data for 2014-2017 bridged-race data) eTable 4. Observed, Estimated Expected, and Estimated Crude Excess Mortality Associated with Alzheimer’s Disease or Related Dementias from March 2020 to February 2022 in the United States, Stratified by Place of Death eFigure 1. Percent Differences in the Number of ADRD Deaths (left panel) and Population Sizes (right panel) Between Single-Race vs. Bridged-Race Data (2018-2020) eFigure 2. ADRD Death Subgroups eFigure 3. Observed, Expected, and Adjusted Observed Death in nursing home/long-term care facilities between 2020/03 and 2022/02, adjusted for percentage changes in population size eFigure 4. Changes in the number of monthly deaths from leading causes of death between 2020/03 and 2022/02 eFigure 5. Changes in State-level Crude Excess Deaths Associated with Alzheimer’s Disease or Related Dementias between Year 1 and Year 2 in the United States, by Maximum Vaccination Coverage Rate and Vaccination Velocity [file jamaneurol-e232226-s001.pdf]

Supplemental Online Content

Chen R, Charpignon ML, Raquib RV, et al. Excess mortality with Alzheimer disease and related dementias as an underlying or contributing cause during the COVID-19 pandemic in the US. *JAMA Neurol*. Published online July 17, 2023. doi:10.1001/jamaneurol.2023.2226

eAppendix 1. Adjusted Observed Deaths based on Changes in the Number of Nursing Home Resident before and after the COVID-19 Pandemic Lockdown
eAppendix 2. Asymptotic Maximum Coverage Rate (K) and Vaccination Velocity Estimation
eTable 1. Alzheimer Disease and Related Dementias ICD Codes
eTable 2. Observed and Estimated Expected Per-Capita Deaths Associated with Alzheimer Disease and Related Dementias Between January and December 2019
eTable 3. Observed, Estimated Expected, and Estimated Excess Mortality Associated with Alzheimer's Disease or Related Dementias from March 2020 to February 2022 in the United States, Stratified by Racial and Ethnic Identity ("imputed" single-race monthly death data for 2014-2017 bridged-race data)
eTable 4. Observed, Estimated Expected, and Estimated Crude Excess Mortality Associated with Alzheimer's Disease or Related Dementias from March 2020 to February 2022 in the United States, Stratified by Place of Death
eFigure 1. Percent Differences in the Number of Alzheimer Disease and Related Dementias Deaths (left panel) and Population Sizes (right panel) Between Single-Race vs. Bridged-Race Data (2018-2020)
eFigure 2. Alzheimer Disease and Related Dementias Death Subgroups
eFigure 3. Observed, Expected, and Adjusted Observed Death in Nursing Home/Long-Term Care Facilities Between March 2020 and February 2022, Adjusted for Percentage Changes in Population Size
eFigure 4. Changes in the Number of Monthly Deaths from Leading Causes of Death between March 2020 and February 2022
eFigure 5. Changes in State-level Crude Excess Deaths Associated with Alzheimer Disease and Related Dementias between Year 1 and Year 2 in the United States, by Maximum Vaccination Coverage Rate and Vaccination Velocity

This supplemental material has been provided by the authors to give readers additional information about their work.

© 2023 Chen R et al. *JAMA Neurology.*

eAppendix 1. Adjusted Observed Deaths based on Changes in the Number of Nursing Home Resident before and after the COVID-19 Pandemic Lockdown

We retrieved the monthly number of nursing home residents from the Centers for Medicare & Medicaid Services (CMS) database for the years 2019 to 2022 (https://data.cms.gov/provider-data/dataset/4pq5-n9py). To ensure consistency across the four years, we limited our analysis to providers that reported resident numbers for all four years, resulting in a sample of 14,969 (~90%) nursing home providers. To handle missing data, we imputed the average number of residents from available months within the same year for each missing month of a given provider. For each year and month between 2019 and 2022 (inclusive), we calculated the total number of residents of all providers. We used the pre-pandemic months of January to December 2019 as reference levels. For each month between March 2020 and February 2022, we compared the total number of residents to the reference level during the corresponding month in 2019. As a summary statistic, we computed the percentage change in monthly population size, relative to 2019. Using this percentage change, we adjusted the observed number of deaths between March 2020 and February 2022 by dividing it by the percentage change of the resident population for that month. For example, the adjusted number of observed deaths in March 2020 was calculated by dividing the observed number of deaths by the percentage change of the resident population comparing March 2020 to March 2019:

$$\frac{\text{Observed number of deaths} * \text{Total number of residents in March 2019}}{\text{Total number of residents in March 2020}}$$

Our approach assumes that the ADRD-related death rates of the population residing in the nursing homes would be proportional to the total number of individuals residing in the nursing home. Additionally, we assume that changes in the population of nursing home residents reported in the CMS data correspond with changes in the population that would be reported as dying in a nursing home in the death certificate data. We believe both assumptions to be plausible. We adjusted for changes in the population size rather than attempting to estimate the exact size of the NH/LTC population (denominator) for two main reasons. First, not all providers consistently reported the monthly number of residents throughout the study period. Therefore, owing to missingness in the monthly time series data, our analysis could not rely on the absolute number of NH/LTC residents. Instead, we restricted our analysis to providers that consistently reporting data throughout the study period and estimated a relative adjustment factor that is less prone to data errors and selection biases. Second, the monthly numbers of observed deaths used as numerators were derived from the CDC WONDER platform, while the monthly evolution of NH/ LTC population size was obtained from the CMS database. Some facilities that might be considered nursing homes when the death certificate was completed might not formally report to CMS. To avoid any data mismatch, we thus relied on a relative time-varying adjustment factor for the NH/LTC population size rather than possibly flawed estimates of monthly denominators.

© 2023 Chen R et al. *JAMA Neurology.*

eAppendix 2. Asymptotic Maximum Coverage Rate (K) and Vaccination Velocity Estimation

We hypothesized that both the velocity with which each state vaccinated its older residents and the asymptotic maximum vaccination coverage influenced infection and subsequent death rates. In each state, we fitted a separate logistic growth model to estimate the speed of vaccination, denoted by *v*, and the asymptotic maximum coverage rate (2 doses among 65 years and older), denoted by *K,* among individuals aged 65 years and older. We evaluated the relationship between yearly changes in state-level excess mortality, vaccination velocity, and asymptotic maximum vaccination coverage. Because vaccination is most relevant in settings where infection is common, we also stratified by tertiles of excess ADRD-related mortality during year 1 of the pandemic

We defined full vaccination coverage as the percentage of adults aged 65 years or older who received two doses of *any* COVID-19 vaccine. The time series were available daily, from March 5, 2021 to February 28, 2022. We extracted the vaccination data from the CDC COVID-19 Data Tracker on July 24, 2022. While the CDC Data Tracker provides information about vaccine deployment from December 13, 2020 onwards, the percentage of fully vaccinated individuals aged 65+ was not available until March 5, 2021. Importantly, we selected full vaccination coverage as the outcome of interest because two doses are supposed to provide greater protection from infection with SARS-CoV-2 compared to one dose only or with at least one dose. We assumed that the cumulative percentage of the 65+ population being fully vaccinated followed a logistic growth curve.[1] That is, the full vaccination coverage in each state started with exponential growth, reached its asymptotic maximum level, and then stabilized. Under this model, the vaccination dynamics of a given state is characterized by three parameters: the percentage of individuals fully vaccinated at baseline (p_0), the asymptotic maximum coverage rate (K), and the vaccination velocity (v). Thus, the logistic growth curve describes the cumulative percentage of individuals fully vaccinated at a given time t (p_t) as a function of the vaccination coverage at baseline (p_0), the asymptotic maximum coverage rate (K), and the vaccination velocity (v).

$$p_t = \frac{K p_0 e^{vt}}{K + p_0\,(e^{vt} - 1)}$$

To estimate the three parameters for each state, we used the *nls* function in R. We leveraged the *getInitial* function to obtain the best initialization vector *(p0, K, v)*. Optimization constraints were added to reflect the range of admissible parameter values, namely: $K \geq 0$ and <= 100%, $p_0 \geq 0$ and $\leq 100\%$, and $v \geq 0$. Of note, the percentage of fully vaccinated individuals was smoothed prior to model fitting using the *cummax* function in R, thereby ensuring that it is monotonically nondecreasing. Due to inconsistencies in data reporting and poor-quality vaccination coverage data, we did not include West Virginia in the analysis.

Reference:

1. Bruckhaus AA, Abedi A, Salehi S, et al. COVID-19 vaccination dynamics in the US: coverage velocity and carrying capacity based on socio-demographic vulnerability indices in California. *Journal of immigrant and minority health*. 2022;24(1):18-30.

© 2023 Chen R et al. *JAMA Neurology.*

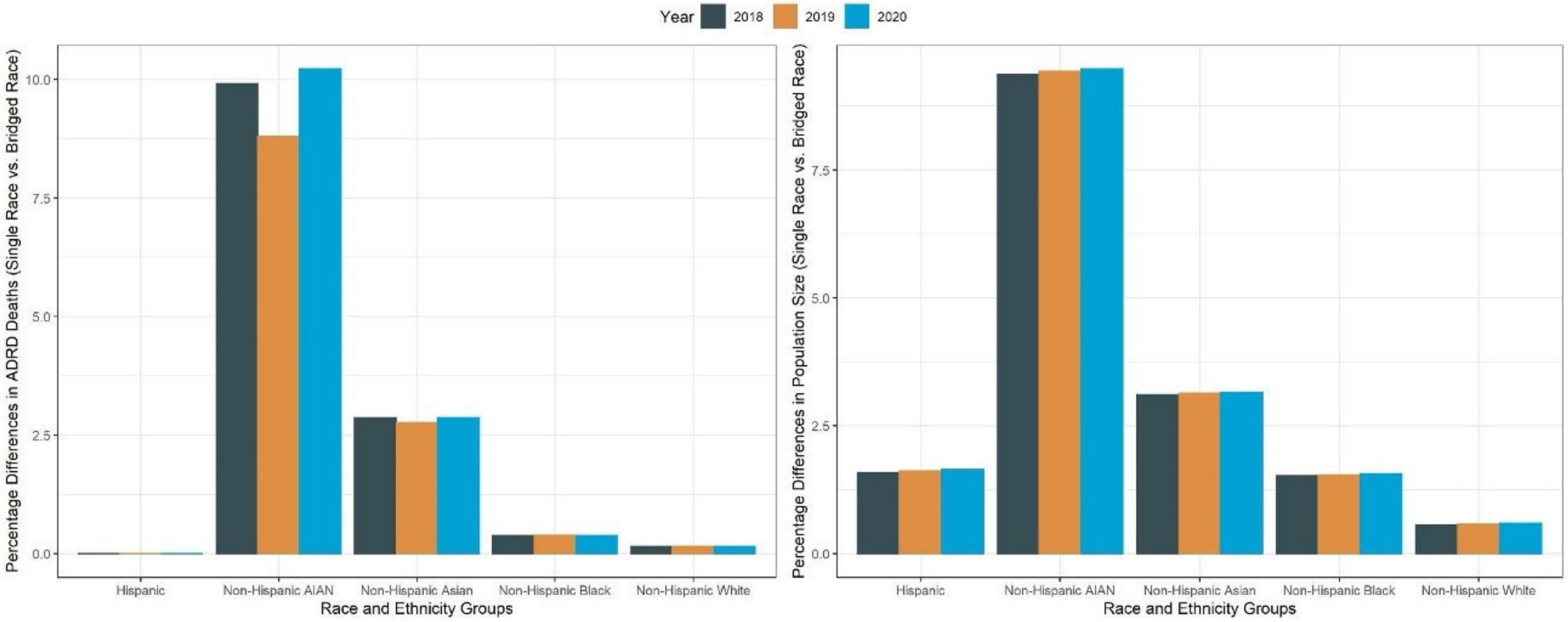

***eFigure 1.* Percent Differences in the Number of Alzheimer Disease and Related Dementias Deaths (left panel) and Population Sizes (right panel) Between Single-Race vs. Bridged-Race Data (2018-2020).** Percent differences were calculated as the number of deaths/population in the bridged race group minus the number of deaths/population in the single race group, divided by the number in the bridged race group**.** AIAN: American Indian and Alaska Native. Single-race and bridged-race death and population data were obtained from CDC WONDER. For comparison purposes, we combined Asian and Native Hawaiian or Other Pacific Islander categories in single-race data in order to replicate the bridged-race Asian or Pacific Islander category.

© 2023 Chen R et al. *JAMA Neurology.*

eTable 1. **Alzheimer Disease and Related Dementias ICD Codes**

ICD code	Types of ADRD
**F01**	**Vascular Dementia**
F01.0	Vascular dementia of acute onset
F01.1	Multi-infarct dementia
F01.2	Subcortical vascular dementia
F01.3	Mixed cortical and subcortical vascular dementia
F01.8	Other vascular dementia
F01.9	Vascular dementia, unspecified
**F03**	**Unspecified dementia**
**G30**	**Alzheimer disease**
G30.0	Alzheimer disease with early onset
G30.1	Alzheimer disease with late onset
G30.8	Other Alzheimer’s disease
G30.9	Alzheimer’s disease, unspecified
**G31**	**Other degenerative diseases of nervous system, not elsewhere classified**
G31.0	Circumscribed brain atrophy
G31.1	Senile degeneration of brain, not elsewhere classified
G31.2	Degeneration of nervous system due to alcohol
G31.8	Other specified degenerative disease of nervous system
G31.9	Degenerative disease of nervous system, unspecified

© 2023 Chen R et al. *JAMA Neurology.*

***eTable 2.* Observed and Estimated Expected Per-Capita Deaths Associated with Alzheimer Disease and Related Dementias Between January and December 2019.**

	Observed Deaths per 100,000 individuals in the population from January to December 2019<br>N	Expected Deaths from January to December 2019<br>N (95 PI)
All ADRD Deaths	771	778 (744, 813)
Age Groups		
65-74 years	32	34 (28, 41)
75-84 years	296	299 (289, 309)
85+ year	2,206	2,196 (2,130, 2,257)
Sex		
Female	811	817 (779, 856)
Male	700	708 (680, 735)
Racial and Ethnic Identity		
White	814	821 (784, 858)
Black	766	771 (739, 803)
Hispanic	572	574 (547, 601)
Asian	392	393 (337, 409)

***Note*.** PI: prediction intervals. Death and population data were obtained from the CDC WONDER. The expected number of deaths was estimated using data from January 2015 to December 2018. Predictions were based on best-fit ARIMA models.

© 2023 Chen R et al. *JAMA Neurology.*

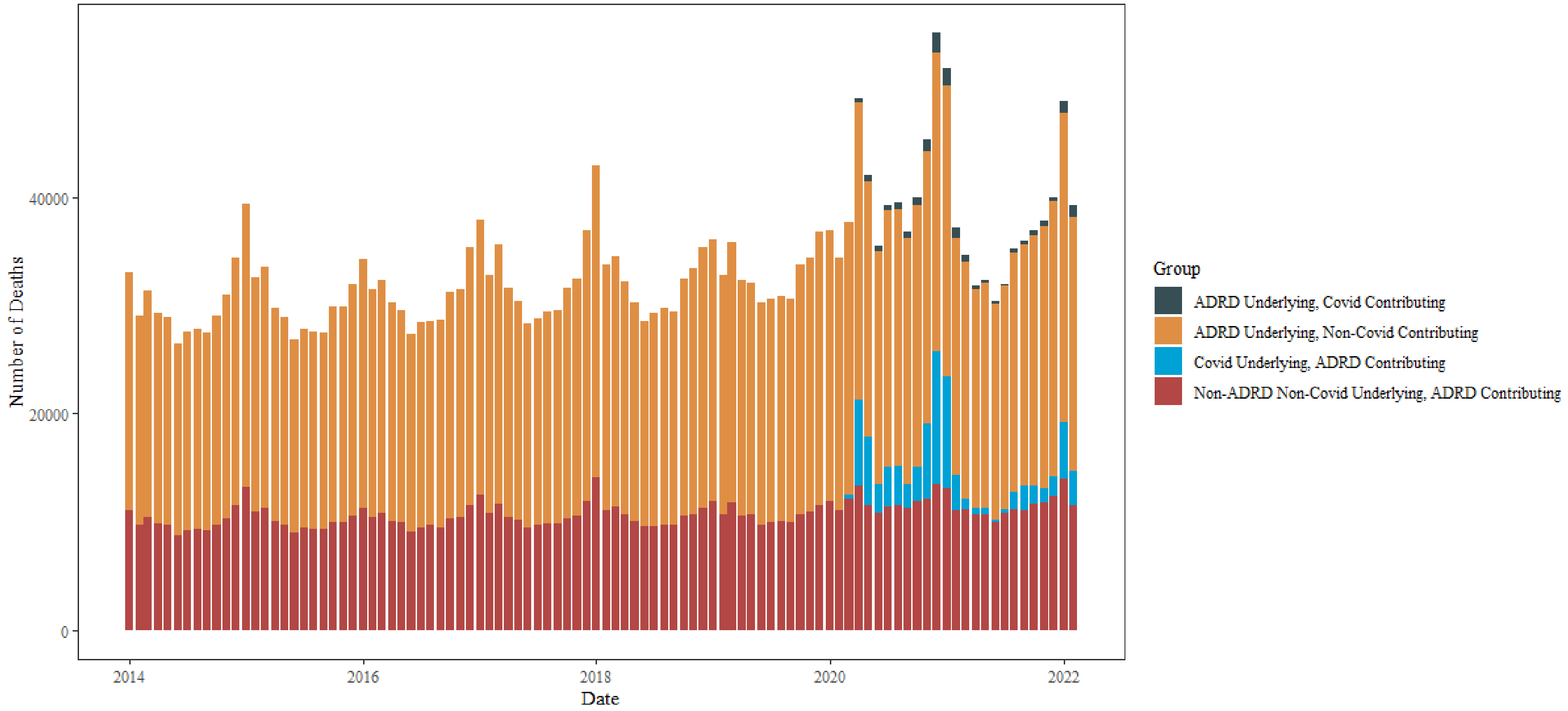

***eFigure 2.* Alzheimer Disease and Related Dementias Death Subgroups.** This figure presents four mutually exclusive groups: 1) ADRD as an underlying cause, COVID-19 as a contributing cause (limed spruce); 2) ADRD as an underlying cause, non-COVID as a contributing cause (yellow); 3) COVID as an underlying cause, ADRD as a contributing cause (blue); and 4) non-Covid and non-ADRD disease (e.g., cancer) as an underlying cause, ADRD as a contributing cause (red).

© 2023 Chen R et al. *JAMA Neurology*.

***eTable 3.* Observed, Estimated Expected, and Estimated Excess Mortality Associated with Alzheimer's Disease or Related Dementias from March 2020 to February 2022 in the United States, Stratified by Racial and Ethnic Identity ("imputed" single-race monthly death data for 2014-2017 bridged-race data)**

	**Pandemic Year 1 (March 2020 to February 2021)**				**Pandemic Year 2 (March 2021 to February 2022)**			
	Observed ADRD-related deaths per 100,000 population	Expected ADRD-related deaths per 100,000 population based on pre-pandemic death rates (95% PI)	Excess ADRD-related deaths per 100,000 population during pandemic year 1 (95% PI)	Observed to expected ratio of ADRD-related deaths in pandemic year 1 (95% PI)	Observed ADRD-related deaths per 100,000 population	Expected ADRD-related deaths per 100,000 population based on pre-pandemic death rates (95% PI)	Excess ADRD-related deaths per 100,000 population during pandemic year 2 (95% PI)	Observed to expected ratio of ADRD-related deaths in pandemic year 2 (95% PI)
Racial and Ethnic Identity								
Asian	570	432 (419 - 445)	138 (125 - 151)	1.32 (1.28 - 1.36)	465	429 (415 - 443)	37 (23 - 50)	1.09 (1.05-1.12)
Black	1,140	835 (817- 857)	305 (284 - 323)	1.37 (1.32 - 1.40)	916	831 (811 - 855)	84 (60 - 105)	1.10 (1.07 - 1.13)
Hispanic	847	625 (614 - 637)	222 (210 - 233)	1.36 (1.33 - 1.38)	690	618 (606 - 631)	72 (59 - 84)	1.12 (1.09 - 1.14)
White	1,096	907 (876 - 938)	189 (158 - 220)	1.21 (1.17 - 1.25)	945	909 (875- 944)	36 (1 - 70)	1.04 (1 - 1.08)

Note. PI: prediction interval. Death data were obtained from the CDC WONDER Multiple Cause of Death Files. Population estimates were obtained from the U.S. Census Bureau Population Estimates Program. Death certificates with any mention of ADRD were included. For each racial and ethnic group, we first calculated the difference between single- and bridged-race death counts by multiplying the number of bridged-race deaths from 2014-2017 by the estimated percent differences over 2018-2020. We then subtracted the difference from the monthly bridged-race deaths, after which we re-ran the ARIMA models using the "imputed" single-race death estimates. All analyses were restricted to aged 65 years and older. Per-capita excess deaths by place of death were not age-adjusted because information regarding the population size by place of death was not available.

© 2023 Chen R et al. *JAMA Neurology.*

***eTable 4.* Observed, Estimated Expected, and Estimated Crude Excess Mortality Associated with Alzheimer's Disease or Related Dementias from March 2020 to February 2022 in the United States, Stratified by Place of Death.**

	**Pandemic Year 1 (March 2020 to February 2021)**				**Pandemic Year 2 (March 2021 to February 2022)**			
	Observed ADRD-related deaths	Expected ADRD-related deaths based on pre-pandemic death rates (95% PI)	Excess ADRD-related deaths during pandemic year 1 (95% PI)	Observed to expected ratio of ADRD-related deaths in pandemic year 1 (95% PI)	Observed ADRD-related deaths	Expected ADRD-related deaths based on pre-pandemic death rates (95% PI)	Excess ADRD-related deaths during pandemic year 2 (95% PI)	Observed to expected ratio of ADRD-related deaths in pandemic year 2 (95% PI)
Nursing Home/Long Term Care	238,624	204,365 (195,947 to 212,805)	34,259 (25,819 to 42,677)	1.17 (1.12 to 1.22)	179,342	201,392 (192,615 to 210,107)	-22,050 (-30,765 to -13,273)	0.89 (0.85 to 0.93)
Home	134,291	99,804 (98,149 to 101,476)	34,487 (32,815 to 36,142)	1.35 (1.32 to 1.37)	132,337	103,533 (101,766 to 105,270)	28,804 (27,067to 30,571)	1.28 (1.26 to 1.3)
Medical Facilities	64,594	46,841 (44,439 to 49,249)	17,753 (15,345 to 20,155)	1.38 (1.31 to 1.45)	53,997	45,441 (42,822 to 48,039)	8,556 (5,958 to 11,175)	1.19 (1.12 to 1.26)
Hospice	31,086	30,151 (29,267 to 31,036)	935 (50 to 1,819)	1.03 (1 to 1.06)	31,218	30,698 (29,772 to 31,615)	520 (-397 to 1,446)	1.02 (0.99 to 1.05)

Note. PI: prediction interval. Death data were obtained from the CDC WONDER Multiple Cause of Death Files. Death certificates with any mention of ADRD were included. All analyses were restricted to aged 65 years and older. To ensure that the results of our stratified analysis by place of death are interpretable and can yield actionable insights, we did not include deaths that occurred in the "Other " and "place of death unknown" settings from the CDC WONDER data.

© 2023 Chen R et al. *JAMA Neurology.*

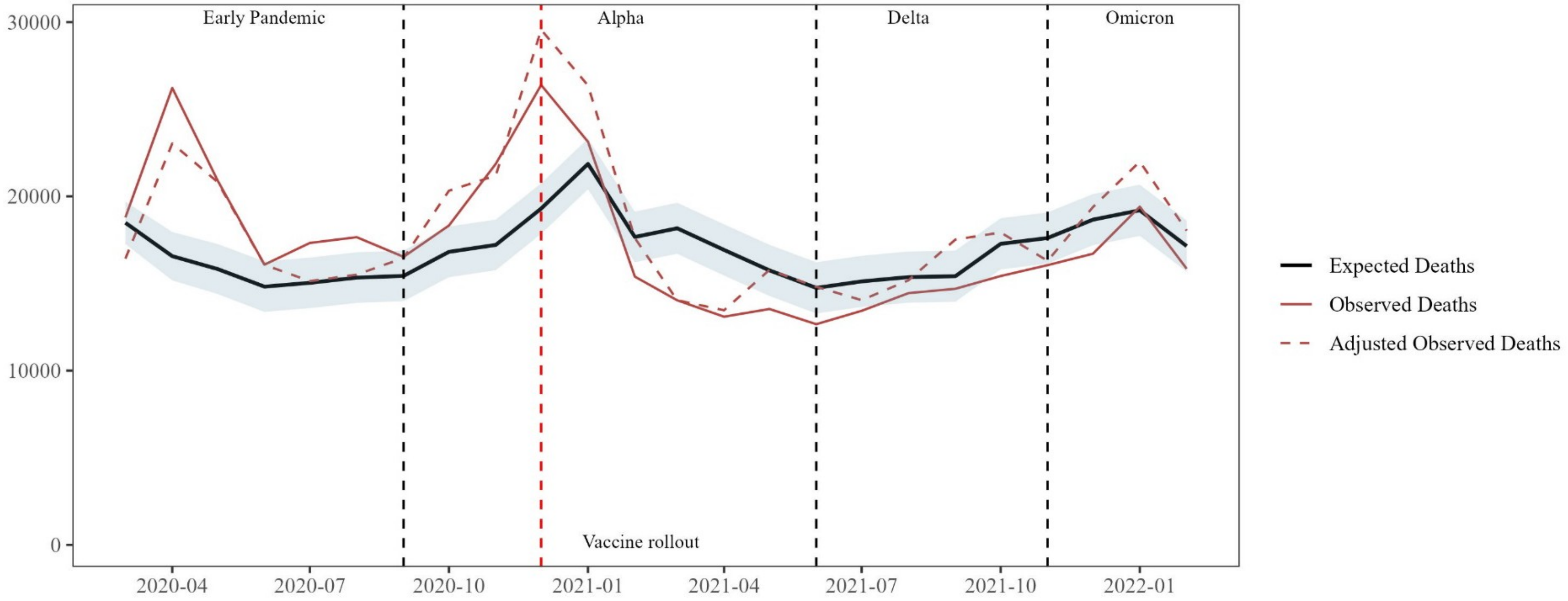

***eFigure 3.* Observed, Expected, and Adjusted Observed Death in Nursing Home/Long-Term Care Facilities Between March 2020 and February 2022, Adjusted for Percentage Changes in Population Size.** Data were drawn from CDC WONDER. We obtained the number of residents of nursing home/long-term care providers for years 2019 to 2022 from the Centers for Medicare & Medicaid Services (CMS).

© 2023 Chen R et al. *JAMA Neurology*.

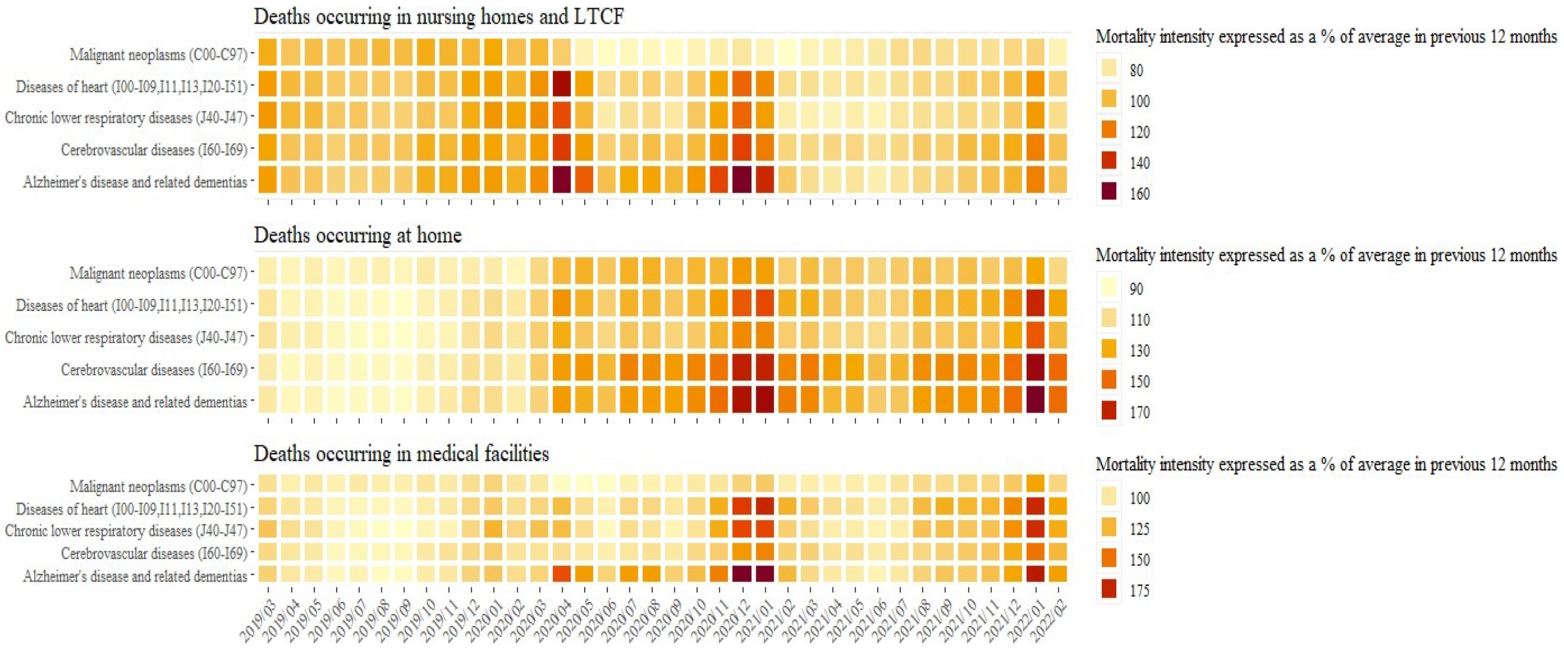

***eFigure 4.* Changes in the Number of Monthly Deaths from Leading Causes of Death between March 2020 and February 2022.** Data were drawn from CDC WONDER. We defined a baseline monthly mortality level for each cause, estimated as the average death count over the 12 months preceding the pandemic (March 2019-February 2020 inclusive).

© 2023 Chen R et al. *JAMA Neurology.*

A.

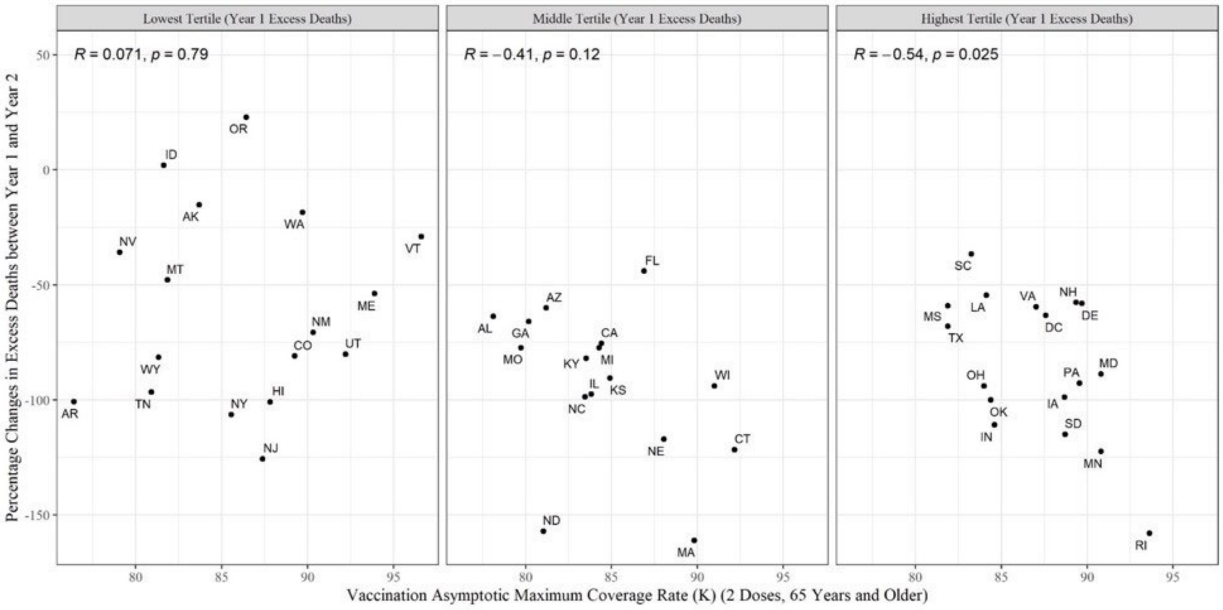

B.

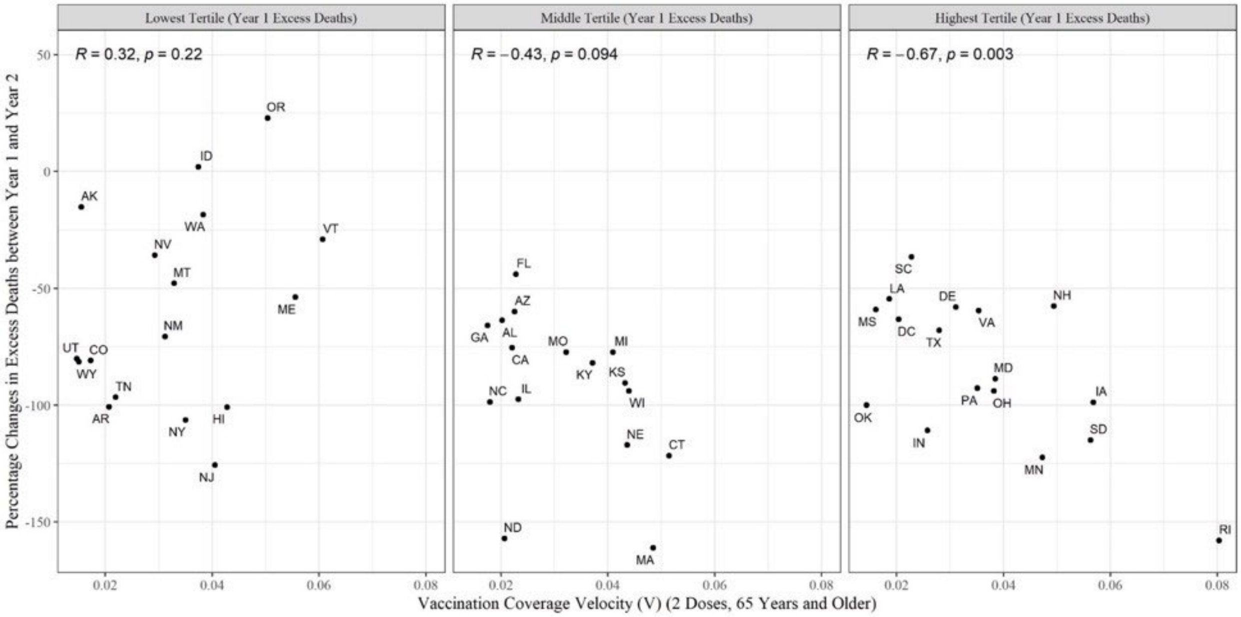

eFigure 5. **Changes in State-level Crude Excess Deaths Associated with Alzheimer Disease and Related Dementias between Year 1 and Year 2 in the United States, by Maximum Vaccination Coverage Rate and Vaccination Velocity.** Death data were obtained from the CDC WONDER Multiple Cause of Death Files. Vaccination coverage rate data were obtained from the CDC vaccination tracker. Changes in excess deaths were calculated by subtracting year 1 excess deaths from year 2 excess deaths, dividing by year 1 excess deaths, and multiplying by 100. Results presented by tertiles of Year 1 excess deaths. We did not include west Virginia due to poor model fit.

© 2023 Chen R et al. *JAMA Neurology.*
