# Supplementary material for: Excess Mortality With Alzheimer Disease and Related Dementias as an Underlying or Contributing Cause During the COVID-19 Pandemic in the US
Source: JAMA Neurol. 2023 Jul 17;80(9):919–28. doi: 10.1001/jamaneurol.2023.2226 (PMC10352932; doi:10.1001/jamaneurol.2023.2226)
Supplement: Supplement 2. — Data sharing statement [file jamaneurol-e232226-s002.pdf]

Data Sharing Statement

Chen. Excess Mortality with Alzheimer Disease and Related Dementias as an Underlying or Contributing Cause During the COVID-19 Pandemic in the US. *JAMA Neurol*. Published July 17, 2023. doi:10.1001/jamaneurol.2023.2226

Data
Data available: Yes
Data types: Data (not involving human participants)
How to access data: The death certificate data are publicly available and can be assessed at: https://wonder.cdc.gov/mcd.html All the code are stored in Github. Once the manuscript is accepted, we will make the code publicly available: https://github.com/2018Ruijia/excess_death_ADRD
When available: With publication

Supporting Documents
Document types: Statistical/analytic code
How to access documents: https://github.com/2018Ruijia/excess_death_ADRD
When available: With publication

Additional Information
Who can access the data: Ruijia Chen: ruijia.chen@ucsf.edu
Types of analyses: We will publish all code for this paper
Mechanisms of data availability: The death certificate data are publicly available and can be assessed at: https://wonder.cdc.gov/mcd.html All the code are stored in Github. Once the manuscript is accepted, we will make the code publicly available: https://github.com/2018Ruijia/excess_death_ADRD
